# Co‐production for service improvement: Developing a training programme for mental health professionals to enhance medication adherence in Black, Asian and Minority Ethnic Service Users

**DOI:** 10.1111/hex.12936

**Published:** 2019-06-27

**Authors:** Iris Gault, Julia Pelle, Mary Chambers

**Affiliations:** ^1^ Faculty of Health, Social Care and Education Kingston University and St Georges University of London London UK

**Keywords:** adherence, BAME, medication, mental health, mental health act

## Abstract

**Aim:**

To co‐produce consensus on the key issues important in educating mental health‐care professionals to optimize mental health medication adherence in Black, Asian and Minority Ethnic (BAME) groups.

**Objectives:**

To identify perceptions of factors enabling or disabling medication adherence. To achieve consensus on content and delivery of an educational intervention for mental health‐care professionals.

**Methods:**

Data were collected from 2016 to 2018. Using individual interviews and a consensus workshop with carers and service users (SUs treated under the 1983 Mental Health Act 1983/revised 2007 for England and Wales), the experience of taking prescribed mental health medication and perspectives on adherence were explored. Data were analysed using 2‐stage qualitative coding via the software tool NVivo version 11 to analyse transcribed data and to produce the main explanatory categories.

**Results:**

SU and carer participants' perspectives substantially altered the original research design. The need to educate students rather than trained professionals was emphasized, and they suggested that educational content should be packaged in a contemporary manner (a virtual reality experience). Findings indicated that education should focus upon understanding the impact of taking prescribed antipsychotic medication on both SUs and carers.

**Discussion:**

The importance of effective communication between health professionals, SUs and carers and a willingness to learn about and appreciate how BAME culture influences perception of mental illness and mental well‐being were highlighted.

**Conclusion:**

In working co‐productively, researchers need to be flexible and adaptable to change.

## 1 INTRODUCTION

People from BAME communities experiencing mental health problems are known to have poorer health outcomes than White service users. Reported to have less incidence of common mental health problems, they are more likely to be diagnosed with severe mental illness, admitted and treated under mental health legislation,^1^ and receive higher dosages of mental health medication.^2^ Contact with mental health services can be fraught, and adherence to prescribed medication is often poor.^3^ Several writers from BAME backgrounds highlight differing cultural beliefs about medication and problems with acknowledging mental health conditions within their communities. They also point out that mental health services could improve their approach to BAME SUs and carers.^4^

The situation is complex, requiring investigation and action across a wide range of issues. A recommendation (supported by evidence of effectiveness) is the use of co‐production and “SUs as experts in shaping services”.^2^ ^,p.15^ Co‐production is recognized and considered as important in researching complicated aspects of health care. The effectiveness of co‐produced initiatives in reducing crisis has been noted.^5^ It is acknowledged that the components of “true” co‐production are debated and can be defined in different ways and at a variety of levels. For the purposes of this study, co‐production is defined as a process in which people who use a service are involved in all stages of the development of a new service or initiative.^6^, ^7^

Co‐produced research is defined as “the collaboration between researchers and others with a stake in a project in its governance, priority‐setting, conducting of research and knowledge translation”.^8^ Nevertheless, it is questionable as to how much “genuinely” co‐produced research exists. Co‐production is not simply a convenient label; “there is such widespread support for the rhetoric of co‐production that we may dismiss…the tensions that arise when professionals and lay people work together”.^8^ ^,p.1^ This study makes considerable effort to take a co‐produced approach but may not meet some of the standards required for “true” co‐production.

## 2 BACKGROUND AND RATIONALE

This study builds on previous studies that explored perceptions of BAME carers for individuals with a mental health condition^9^ and the experience of compulsory medication taking following problematic adherence.^10^ Recommendations from these studies indicated the need to investigate factors that might improve the carer and service user (SU) experience of mental health medication taking from the perspective of BAME communities.

Poor mental health medication adherence and missed contact with mental health services can make relapse more likely. In the United Kingdom (UK), approximately thirteen per cent of suicides in England followed non‐adherence with medication and BAME SUs constituted six per cent of that number. Forty‐eight per cent of all mental health service user homicides across all UK countries were non‐adherent with medication or had lost contact with mental health services. Twenty per cent of those were from a BAME group. When looking at physical health issues, BAME SUs, who died suddenly, represented sixteen per cent of all of those with sudden unexplained deaths.^11^

The Department of Health^12^ calls for better therapeutic alliances between health‐care staff and SUs. Black, Asian and Minority Ethnic groups constitute only 3.5% of the British population and 2.7% of mental health SUs in England. Nevertheless, compulsory treatment under the English and Welsh Mental Health Act^1^ revealed the highest rate ever recorded in 2015/16 with BAME groups forming the largest group at 272.1 per 100 000 Black people. Only 67 out of 100 000 White people were detained under mental health legislation.^13^ Interventions to improve care for BAME groups have included extra health‐care training on cultural competence. It is suggested that this approach has been less than effective, simply making mental health professionals feel uncomfortable and doing little to improve care and/or medication adherence.^2^

Studies report similar issues in many countries where disproportionate numbers of BAME SUs are compulsorily admitted.^14^ An investigation of paths to compulsory treatment in Toronto found that BAME groups were more likely to experience admission via mental health law and less likely to engage with treatment.^15^ A retrospective chart review was undertaken for SUs with psychosis from 6 different ethnic groups (East Asian, South Asian, Black African, Black Caribbean, White European, and White North American) with a sample size of 765. Logistic regression models explored pathways into care. Those from East and South Asian backgrounds were most liable to undergo compulsory admission and treatment despite having overall lower rates of psychosis. The study was limited in that it was not designed to explain relationship or causation; however, its strength was in specifying ethnic minority category in detail.

A phenomenological study found that BAME SUs on community treatment orders (CTOs)1A section of mental health legislation that imposes compulsory medication taking for SUs living in the community. perceived themselves to be receiving additional resources due to being on a CTO. Improved care was appreciated feelings of stigmatization and coercion were experienced. Limitations included this being a small, qualitative study, the sample did not reflect all minority groups, and communication may have been compromised due to lack of interpreters.^16^

A qualitative study on shared decision making found that BAME African American veterans had difficulty in obtaining satisfaction from their mental health service providers. Many factors were influential, including increased anxiety throughout the encounter with professionals. Recommendations were that service providers improve understanding of African American perspectives and implement better communication measures. This was a small, qualitative study, focusing on one medical centre and therefore not intended to be generalizable to a wider population. However, it highlights the poorly explored perspectives of African American veterans experiencing mental health issues.^17^ The requirement to better understand BAME perspectives is supported by another qualitative study into the impact of stigma on help‐seeking behaviour in mental health. The complexity of spiritual beliefs, stigma and the need for improved co‐produced work on service improvement was stressed.^18^

The literature indicates over‐representation of certain BAME ethnic groups within compulsory pathways into care. 12, 14, 15 Studies into the problem of poor medication adherence and service engagement for BAME SUs stress the need for more in‐depth understanding of the perspectives of those involved.

## 3 METHODS

**Figure 1:**
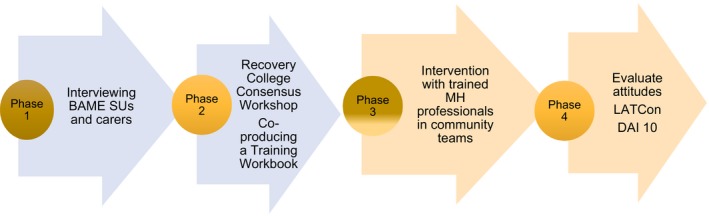
Original plan. LATCon = Leeds Attitude Towards Concordance scale.^28^ DAI 10 = Drug Attitude Inventory 10. Nielsen, R., Lindstrom, E., Nielsen, J. and Levander, S. (2012) DAI 10 is as good as DAI 30 in Schizophrenia. 22 pp. 747‐750

**Figure 2:**
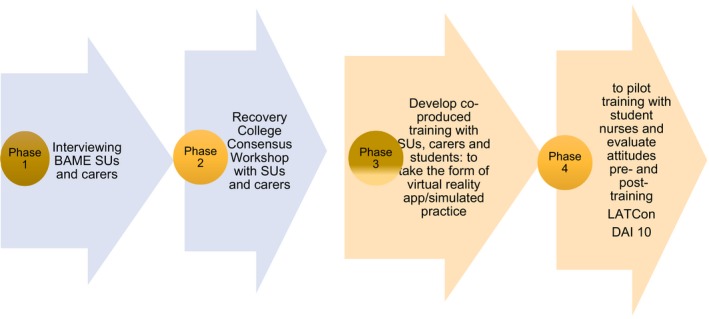
Revised Plan. LATCon = Leeds Attitude Towards Concordance scale.^28^ DAI 10 = Drug Attitude Inventory 10^29^

This paper describes phases 1 and 2 of a 4‐phase study (see Figures 1 and 2 to indicate how participants re‐shaped research design) and aimed to examine how service improvement regarding medication adherence can be achieved by working with BAME SUs and carers. The intention was to hear their perspectives on medication adherence and gain consensus on an initiative to enhance professional effectiveness in this area. Consequently, a qualitative approach was selected, initially interviewing mental health SUs and carers from a BAME background to obtain rich, in‐depth perceptions and interpretations of mental health care and medication taking; following analysis of the interviews, a consensus workshop was conducted with interview participants to reach agreement on the most important areas in which to educate professionals.

### 3.1 Aim

To co‐produce consensus on the key issues important in educating mental health‐care professionals to optimize mental health medication adherence in Black, Asian and Minority Ethnic (BAME) groups.

### 3.2 Objectives

**Phase 1.** To identify perceptions of factors enabling or disabling medication adherence.
**Phase 2.** To achieve consensus on content and delivery of an educational intervention for mental health‐care professionals.

#### 3.2.1 Phase 1. Individual interviews

Phase 1 focused on open‐ended interviews, sampling theoretically and analysing data using a staged thematic approach. It considered participants' perspectives on key elements enabling or disabling medication adherence.^19^ Data collection was undertaken by 1 member of the team. Analysis was conducted in partnership with the team, including SU members from the peer‐led Recovery2The first Recovery Colleges developed over 8 years ago in the UK. They are growing across England, some European countries, Australasia and Japan. ‘For people recovering from a period of mental ill health, getting involved in the local community can be an important part of their recovery, but finding services that fit with their lives can be difficult. An increasingly popular option is to enrol at a recovery college, where they can study in an environment geared towards helping people in their ongoing recovery from mental illness.' https://www.mentalhealthtoday.co.uk/recovery-colleges-bridging-the-gap-in-mental-health-service-provision p.1. College. Data were voice‐recorded and transcribed. Analysis proceeded simultaneously with the iterative process directing the sampling. Analytic processes were guided by an adapted version of the initial two stages of the grounded theory technique. Many agree that the use of a systematic coding process adds transparency and credibility to qualitative methods.^19^, ^20^

#### 3.2.2 Phase 2. Consensus workshop

To validate findings and reach consensus on content for the training workbook for community mental health professionals, findings from phase 1 were presented to a group of SUs and carers (3 SUs, 2 carers (who had participated in interviews).

### 3.3 Participants and sampling

The study recruited 10 BAME SUs and 5 carers with all given the option of being interviewed and participating in the follow‐up consensus workshop (See Tables 1, 2, 3, 4). Three service users and two carers agreed to both be interviewed and engaged with the consensus workshop. Although the quantity of participants is not the major concern in qualitative work, it is necessary to continue sampling until saturation is established.^21^ Sampling therefore proceeded theoretically, selecting those indicated by the emerging themes. Participants volunteered in response to posters advertised in the local Recovery College resulting in the sample size of 15. It should be noted that recruitment to research projects from BAME groups can be difficult; BAME people form part of those “hard to reach” groups due to several factors, not least, a history of mistrusting mental health services.^18^ ^,p.375^

**Table 1:** Demographic characteristics: Service users

Male	Female	Ethnicity		Age	
3	7	Black African Black Caribbean Asian	2 5 3	18‐30 31‐40 41‐50 51‐60	5 4 1 0

**Table 2:** Demographic characteristics: Carers

Male	Female	Ethnicity		Age	
2	3	Black African Black Caribbean Asian	2 2 1	18‐30 31‐40 41‐50 51‐60	1 1 3 0

**Table 3:** Participant details

Participant 1	18‐year‐old Asian male service user
Participant 2	30‐year‐old Asian female service user
Participant 3	22‐year‐old Black African male service user
Participant 4	50‐year‐old Black Caribbean female service user
Participant 5	42‐year‐old Black Caribbean female carer
Participant 6	38‐year‐old Black African male service user
Participant 7	30‐year‐old Black Caribbean female service user
Participant 8	28‐year‐old female Asian service user
Participant 9	27‐year‐old Black Caribbean female service user
Participant 10	34‐year‐old Black Caribbean female service user
Participant 11	27‐year‐old Black Caribbean male service user
Participant 12	44‐year‐old Black African female carer
Participant 13	36‐year‐old Asian male carer
Participant 14	29‐year‐old Black African female carer
Participant 15	44‐year‐old Black Caribbean female carer

**Table 4:** Demographic characteristics consensus workshop

Male	Female	Ethnicity		Age	
1	4	Black African Black Caribbean Asian	3 2 0	18‐30 31‐40 41‐50 51‐60	1 3 1 0

#### 3.3.1 Ethics

The study was submitted to and gained approval from the NHS Trust ethics committees and the national Health Research Authority (HRA)^22^ (2 NHS sites and the Health Research Authority) and was adherent to all HRA procedures. Potential participants received a letter with information about the purpose of the study and about participating in the study. Interviewees were assured of confidentiality. A consent form accompanied this with potential participants given two weeks to sign and return. All data were rendered unidentifiable via a coding system. Digital voice recordings were available only to the research team and kept in a locked cupboard on university premises for up to 10 years. These are accessible only on password‐protected memory sticks.

Participants were assured that interviews would be terminated at their request at any time. Interviews were conducted in the Recovery College building to ensure that support could be arranged should anyone require this. Service users/carers may inadvertently disclose information regarding misconduct that would require the researcher (under their professional code of conduct) to report to the appropriate authorities. This information was made clear to all participants at the commencement of interview, so they could monitor what they wished to say.^23^

#### 3.3.2 Inclusion criteria for interview

Black, Asian and Minority Ethnic (BAME) SUs and/or carers with current or recent experience of mental health medication and treatment under the Mental Health Act (1983/revised 2007 for England and Wales).

#### 3.3.3 Exclusion criteria for interview

Ethically, it would be unsound to include people who are actively distressed. Therefore, although people on mental health sections were included, this only occurred when both SU and carer agreed that participation was unlikely to be harmful. In addition, this study only targeted adult SUs (over 18 years of age).

### 3.4 Data generation

The semi‐structured interview guide based on the best practice guidance from Royal College of Pharmacology (see Appendix 1) was piloted with one carer and one SU. The research team agreed that the interview guide was appropriate. Interviews were conducted by (X), analysed by (Y) and (Z) (the other faculty research team members) and considered with SU members of the Recovery College. As qualitative methods based on grounded theory, interviews became less structured over time, following topics of interest developing. They took approximately 30‐40 minutes and were conducted in the Recovery College (as this was a supportive environment for service users and carers), and all were digitally recorded and transcribed by the team.

### 3.5 Data analysis

#### 3.5.1 Phase 1

**Figure 3:**
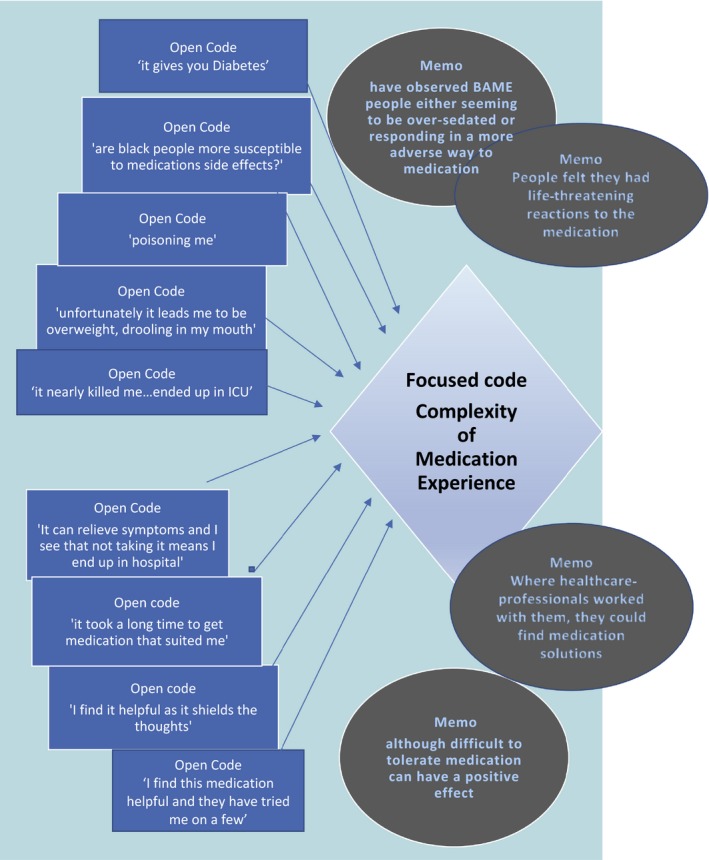
Example of 1st and 2nd stage open and focused coding leading to explanatory category

Grounded theory, originally developed in the 1960s by Glaser and Strauss, has been the topic of much debate ever since with even the archetypes revising their own work and disagreeing with one another. This study accepts the views of contemporary researchers such as Charmaz^21^ that despite debate, the constant comparative technique of 2‐ or 3‐stage coding allows for better levels of transparency in analysis. Following transcription, data were analysed using a 2‐staged coding process based upon the stages of grounded theory.^21^, ^24^ For the purposes of this study, it is considered that qualitative research is enhanced by the use of systematic coding that can illustrate how the data move from in vivo sections of text to become explanatory categories (in grounded theory, the term “category” is used rather than “theme”).^20^, ^24^ The NVivo 11 computer program enabled the handling and coding of large amounts of text, and it is argued, enables greater transparency of the analytical process.^24^ Transcriptions were scrutinized line by line to fracture the data and identify multiple open codes. Descriptive memos were assigned to add depth to codes, and using3Original grounded theory calls this stage “axial coding”. Charmaz^21^ who has further developed grounded theory coding techniques terms this stage ‘focused coding’ as a stage prior to axial coding. She indicates that open and focused coding is sufficient for many projects and in the phases 1 and 2. As described in this study, we take this approach. focused coding (as Charmaz labels 2nd stage coding), open codes are merged and connected with others to form explanatory categories^24^ (see Figure 3 for an example). As noted above, findings were considered with Recovery College service user colleagues and respondent validation took place, checking out findings with certain participants where clarification was required.^21^ As will be discussed at a latter stage, participants did challenge researchers' assumptions.

#### 3.5.2 Phase 2

Five interviewees (three SUs and two carers) further volunteered to meet to consider the findings from the interviews in a consensus workshop format. Consensus workshops, using nominal group technique, are recommended as a practical method for establishing agreement in areas that may lack objective evidence and has been established as effective in design of health‐care education.^25^ Nominal group technique was employed to present and rank findings. The main findings of the interview process were not changed but participants reflected upon these, shared ideas, debated, challenged, shaped and ranked the most important elements of the findings for education. They arrived at consensus on content and mode of delivery of the educational initiative to enhance professional understanding of BAME perspectives on prescribed antipsychotic mental health medication. The consensus workshop also challenged and changed researchers' ideas about the target audience.

### 3.6 Findings Phase 1. Individual Interviews

Initial open coding produced hundreds of in vivo codes focusing mainly on what was perceived as poor professional communication on medication, little concern for physical health, for side‐effects, the role of family, both helpful and unhelpful, cultural misunderstandings and miscommunication. Second stage‐focused coding then connected open codes into explanatory categories.^26^ Using descriptive memos, to add depth and context, explanatory categories were constructed by comparing various open codes, determining where they describe similar issues, noting associations between codes and assigning an overall descriptive or conceptual label (See Figure 3).

Following 2nd stage‐focused coding, three main explanatory categories emerged to describe the process as perceived by participants on their experience of mental health medication adherence. These three categories told a story of a complex experience with medication, feeling unheard and an overall perception that their situation was poorly understood.

### 3.7 Complexity of BAME medication experience

**Figure 4:**
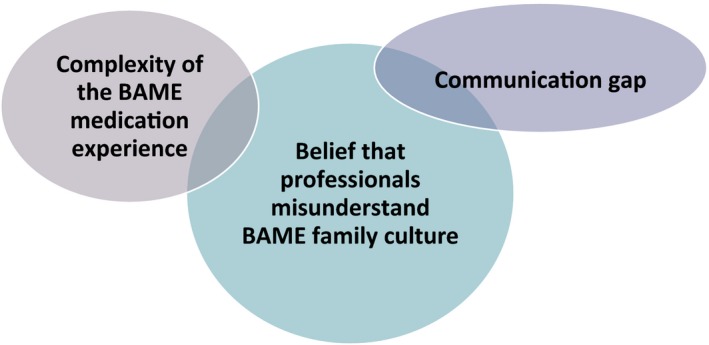
The explanatory categories

The medication experience was described as complex and multi‐faceted. Examination of the data revealed multiple instances of participants' anxieties regarding medication yet also a clear perception that it was necessary (Figure 4). Service users expressed concern at the mental and physical effects of medication and noted they had observed BAME people (to appear) to experience more discomfort than White SUs.

(P = participant, SU = service user, C = carer see Table 3).

SUP.7 “maybe people from an African or Caribbean background are more susceptible?”….
SUP.3 “is there anything on BAME people having worse reactions?”
SUP.1 “I definitely saw BAME people looking worse than others”, and
Others SUP.6 described experiencing medication as “Feels like over sedation ‘zombified’ and unfortunately it leads me to be overweight, drooling in my mouth and it makes me like …sedated and knocked out”
CP.15 described how her relative had “weight gain, drowsiness and very low motivation…now she has Diabetes”

The concern was not merely about the discomfort of experiencing medication side‐effects but at the fact that many had developed conditions such as diabetes and/or cardiac complications. However, despite finding the medication experience uncomfortable and potentially physically dangerous, all acknowledged that not taking medication leads to worse outcomes.

SUP.6 “I find it helpful because it shields the thoughts”
SUP.7 “I don't really like it but I know I need it now”
SUP. 1 “It can relieve symptoms and over time I see that not taking it means I end up in hospital….I had really scary visions….I didn't like that”
CP.15 “when she's on the medication she stays well for a while but then she stops taking it”

Their experience was that it was difficult to arrive at a situation where they could get access to medication that was helpful.

CP.12 “it is difficult to get to a stage where the medication suits him”
SUP.2 “I would prefer medication that is tolerable, it took a long time to get to medication that suited me”

Therefore, medication adherence or non‐adherence was never a simple case of not wishing to take the pills. It was a complicated and anxiety‐provoking process where despite acceptance that medication helped prevent psychotic symptoms, there was real concern that it also caused physical harm. This complex situation could be helped or hindered by their communication with mental health professionals.

### 3.8 The communication gap

Carers and SUs often found their encounters with professionals to be less than helpful. Some observed that despite speaking in English, communication failed for many reasons. Service users felt unheard and powerless in many situations.

SUP.2 “they speak in a different language… we don't understand each other”
SUP.7 “it gets lost in translation”
SUP 8 “I don't honestly think they want to listen to me”…“my suggestions for medication are not taken on board”

There was also a perceived need to behave and communicate in a certain way when seeing a mental health professional.

SUP.6 “Because I have to be like… I have to be quiet. How do I say? I have to be quiet like not be arrogant or rude to them, behave myself so I get the right treatment, I am nice and using the language”
SUP.6 had observed others “being themselves” ie. loudly expressing concern or dissatisfaction at their treatment… and how this behaviour could lead to a professional perception of potential violence with adverse consequences of treatment under the mental health law/injected against their will.
SUP.6 “I have been to other courses where they say, where they are training us (SUs) how to access the care more fully…..they show you how to communicate with the professional so you get the best treatment”
SUP.10 “I get on well with them now but I've been ill for over 20 years and they know me now”
SUP. 9 “I like it here (a rehabilitation hostel), my key worker does make time to listen to me but over the years I've had shocking treatment where no‐one listened”
CP.5 “communication is the key thing”
CP.14 “they think she's going to be violent as she prays loudly and shouts”
SUP.6 observed that one of the most useful courses at the local Recovery College is on how to communicate with mental health professionals in order to achieve best treatment/medication outcomes. This theme was reflected by many participants as they felt they had to behave in the manner expected by health professionals. “Being themselves” in many BAME cultures could mean being loud and direct in communication. It was noted that this communicative behaviour could be misinterpreted by health‐care professionals and perceived as dangerous when in fact, it was simply loud.

### 3.9 Belief that professionals misunderstand BAME family culture

This communication gap was attributed to a belief that health‐care professionals (even if BAME themselves) did not understand BAME SUs who had grown up in (often) deprived circumstances in a large city. Participants expressed views that there was little professional appreciation of the behaviours and family backgrounds of those from an inner city BAME experience. There was limited understanding of the impact of culture and religion.

CP.15 “Most of the professionals are white or black African. There are black African doctors and nurses but they don't come from the same places as us”……“if you end up being a doctor, it's not very likely you come from my background…how can they understand what it's like for me and my relative?”…“African nurses seem to expect a certain type of behaviour….not very sympathetic to illness”
SUP.4 “Doctors tend to come from a place of privilege…how can they judge what is appropriate behaviour for me? ”
SUP.5 “it's also about our social experience. We come with a triple whammy of stigma. Society stigmatises mental illness, too many of the professional do it but we also have the older family members. They see it as bad behaviour or punishment for bad behaviour”
SUP.8 “It's family denial: Family is important and families often do not understand mental ill health; they may see it as shameful or simply as bad behaviour or a consequence of bad behaviour such as drug misuse….you do not bring that through your parents' door”…a disgrace, a shame
SUP.11 “my family thought it was payback for me doing bad things…they don't see it as illness…and I was involved in some bad things as a young man growing up round here”
SUP.1 “my family don't really understand…they think I'm playing up but those voices and visions were really scary…I can't live at home as they don't think I should be on the tablets”
SUP.3 “I've experienced some awful things through my family…they don't believe in mental illness. They have not been supportive at all and the doctor never asks about that”
SUP.2 “my family tried to lock me up…they tried to get me married off but the other family found out about my mental illness and called it off. My father was furious. I've had my things stolen from me. They won't have anything to do with me and my husband now”
CP.13 “the family are ashamed. They don't want to believe it”
CP.14 “no health care professional has actually had a conversation with her about how she feels versus against the differences, it's dismissed. When she says I hear God or I am hearing voices” the family are not going to attribute that to an illness, they are going to attribute that to “well let's try and get you delivered… My church is predominantly Nigerian and African descents, we talk about spiritual things all the time and we pray against any kind of demonic interference so it's not an unknown/unheard of thing to that in our culture, but I don't know, maybe because most of the doctors are western they can't really relate or understand… they kind of dismiss it down to the illness, and some of it is… I would definitely recommend doctors and professionals that are dealing with anyone, a service user, with mental illnesses to really understand their upbringing, try and understand why they have these thoughts.”

Participants perceived that SUs from BAME backgrounds had an extra layer of difficulty in comparison to others. Not only was there a communication gap with health‐care professionals who lacked appreciation of their cultural behaviour; BAME families (especially older members) tended to deny mental disorder, regarding it as bad behaviour to be addressed through better discipline and/or prayer. They believed that these issues were more prevalent in BAME families and that professionals needed more understanding of their family backgrounds. Addressing cultural beliefs was seen as important in understanding the situation for the service user.

### 3.10 Consensus workshop

Five participants (three SUs and 2 carers, see Table 4) agreed to be involved in the consensus workshop. The purpose was (a) to validate (or challenge) findings and (b) to consider how these could be used to develop an educational intervention to enable better professional understanding of BAME perspectives on mental health medication adherence. Questions and key points are presented in Table 5. Participants were asked to rank each category in order of importance and then debate potential educational approaches. They were unanimous that the two categories: *Complexity of the BAME medication experience* and *The communication gap* should be subsumed under category 3: *Belief that professionals misunderstand BAME family culture*.

**Table 5:** Consensus group questions

Questions	Key points
Do you agree that complexity of the BAME medication experience is important?	Yes it is important but it's part of the wider experience
If so, what are the important aspects for an educational intervention?	C M Healthcare professionals would need to feel how it is for service users and carer
Is it more, less or of equal importance to the other findings?	SU E It's important but it is part of something bigger…it links with the others
Do you agree that the communication gap is important?	C M Yes it can be massive, “lost in translation” ‘though we all speak English, it's like we speak different languages
If so, what are the important aspects for an educational intervention?	SU L You can't just tell people… you have to show them
Is it more, less or of equal importance to the other findings?	C M Again, it's linked….part of the bigger picture
Do you agree that the perception that healthcare professions misunderstand BAME culture is important?	All Yes it is absolutely key. It's about everything, not just medication but class, background, family…
If so, what are the important aspects for an educational intervention?	
Researcher “what about education for the family? Should services be doing more to help families understand what mental illness is and its effects?”	Laughter from workshop participant SU M “you do that it if makes you feel better but they'll never change….not our parents” generation……it might change down the line but we're stuck with this for now…….it would be good if the professionals understand that’.
Researcher “what about education for the family? Should services be doing more to help families understand what mental illness is and its effects?”	SU L (with nods and gestures indicating of approval from all the others) “you have to start with the students….there's no point doing it with people already qualified for a while”
Researcher “so not a training booklet for community teams?”	SU E “again, if that's what you want to do, then do it but you really want something for the younger ones……….something with impact….you need to target those in training” C M “an immersive experience” SU S “something that helps them FEEL what it is like to experience being treated as less equal, as not being heard, as being thought of as violent when actually we are just naturally loud”
Is it more, less or of equal importance to the other findings?	It covers everything…the other two findings are part of this wider picture.

Participants agreed that any educational content for mental health professionals needed to include (a) knowledge of different BAME cultures (acknowledging there is no simple and singular BAME culture), (b) how culture influences both the experience of medication taking and the problematic communication, (c) the role of family in caring for relatives with a mental illness and (d) the socio‐economic factors which impact on the development of mental illness.

Participants clearly challenged the original intentions of the study (see Table 5), particularly around the delivery of content and to whom it should be addressed. They indicated the need to produce contemporary and impactful methods of delivery (not a booklet) and emphasized that education works most effectively with younger people, suggesting student health‐care professionals should be the focus. It was suggested that the next steps in the project (to be reported in phases 3 and 4 of this study) should involve working with students (BAME where possible). The content should be delivered via a contemporary and impactful method such as virtual or simulated reality.

## 4 DISCUSSION

Results from this study resonate with previous findings in the literature in that the experience of taking medication is perceived as complex and difficult. The negative effects of the medication experience can be exacerbated by what is perceived as a communicative gap between service users and professionals. Conversely, where professionals communicate well and listen to SUs and carer concerns, the medication experience can be helpful. As previously noted, there can be perceived benefits when SUs and professionals are able to establish better communication and provide adequate resourcing.^16^ Communication is a major issue, and participants appreciated professionals listening and demonstrating they heard the medication concerns of SUs and carers. Often, this would occur after a prolonged time and contact with mental health professionals, pointing to the importance of continuity of care. The time factor and need for continuity of contact with professional carers have been noted within the literature. A study researching clinician perspectives on men who identified as “Black” found that the clinicians perceived the therapeutic relationship as improving over time.^27^

However, the most powerful category, as agreed within the consensus workshops, was that of the poor understanding of BAME culture. This lack of understanding was perceived to overshadow all other issues. The medication experience and how professionals communicated was perceived as being influenced by whether the professional had understanding of BAME culture.

It was agreed that “BAME culture” was not an easily homogenous entity and was instead, complex and multi‐layered.^15^ However, that is not a reason to ignore the reality of differing cultural needs and expectations. Participants expressed the view that medication effects and side‐effects might be more potent for them, leading to poor physical health; their perspective was that they were denied talking therapies and instead had medication prescribed to control them. They believed that they were seen as potentially violent or troublesome. Additionally, their perception was that MH professionals had little in common with them and possessed limited understanding of the powerful effect of stigma in BAME families and communities. As noted in the literature, mental illness and stigma in BAME (African and African descent in their study) groups represent a “triple jeopardy in terms of stigma”: self, family and community.^18^ ^,p.381^

As stated earlier, co‐production in this study was valued as a guiding principle. However, there is no doubt that the extent of “true” co‐production could and should be improved and deepened. In this study, the local Recovery College provided guidance and advice. As discussed above, the latter phases where findings were considered with participants in consensus workshops highlighted how SUs challenged researchers' intentions and assumptions about how education should be delivered and suggested that the study's target audience should be student mental health‐care professionals. In doing so, they changed the process and outcomes of the study. MIND^5^ note that co‐produced research is less likely to produce stigmatized findings.

## 5 CONCLUSION

Policy calls for better therapeutic alliances between mental health professionals and SUs and carers from BAME backgrounds. However, statistics on compulsory treatment and the explanations provided by BAME participants in this study demonstrate that the experience of mental health medication can be improved. Most striking is the perception that health professionals (from backgrounds dissimilar to the BAME participants in this study) have limited appreciation of the impact of culture on taking prescribed antipsychotic medication and the BAME experience of care. This study's main intention was to take a co‐produced approach to genuinely hear SU and carer perspectives on mental health medication and views on adherence. Nevertheless, BAME SUs and carers debated and challenged findings (possibly influenced by professional assumptions such as educating elders), illustrating the depth of the gap in understanding. As noted earlier, in both policy and research, there can be little discussion of working with SUs in a manner that addresses and acknowledges that inevitable tension or disagreement will arise.^8^ If health professional education is to be improved on BAME perspectives on mental health, researchers and health professionals will need to be prepared to hear what is being said.

## 6 LIMITATIONS

This is a small‐scale qualitative study that makes no claim to generalize. All findings relate specifically to the participants within this research.

## 7 RECOMMENDATIONS FOR FUTURE WORK

The final 2 phases of this study will aim to develop, deliver and evaluate a training programme in co‐production with SUs, students and carers making use of relevant contemporary technology.

## Data Availability

The data that support the findings of this study are available from the corresponding author upon reasonable request.

## APPENDIX 1

### TOPIC GUIDE QUESTIONS FOR SERVICE USERS AND CARERS

#### FOR SERVICE USER AND CARERS

Experience of taking compulsory mental health medication or being the carer of someone taking compulsory mental health medication

#### FACTORS WERE HELPFUL IN TAKING THE MEDICATION AS PRESCRIBED?

Did the practitioner (s) you met with; discuss your experience of taking your medication? If yes, what was discussed?

Did the practitioner (s) you met with; discuss how to safely take the medication you were prescribed? If yes, what was discussed?

#### POTENTIAL TO BE MORE HELPFUL

Were there any opportunities missed by practitioners to support you with the taking of your antipsychotic medication? If so, what were these?

#### WHAT TYPE OF SUPPORT DO YOU CONSIDER TO BE HELPFUL FOR PEOPLE FOR THOSE WHO TAKE MENTAL HEALTH MEDICATION OR CARE FOR THEM?

Did the practitioner (s) you meet with; ever signpost you and your carer to additional support to take your medication?

What types of information do you consider to be helpful in enabling people to take medication as prescribed?

**Date. 30/7/17 Study Number: 159657. Topic Guide (based on Royal Pharmaceutical Society Guidelines 2013) version 2.**
